# Conservation priorities of genetic diversity in domesticated metapopulations: a study in taurine cattle breeds

**DOI:** 10.1002/ece3.39

**Published:** 2011-11

**Authors:** Ivica Medugorac, Claudia E Veit-Kensch, Jelena Ramljak, Muhamed Brka, Božidarka Marković, Srđan Stojanović, Hysen Bytyqi, Ljupche Kochoski, Kristaq Kume, Hans-Peter Grünenfelder, Jörn Bennewitz, Martin Förster

**Affiliations:** 1 Chair of Animal Genetics and Husbandry, The Ludwig-Maximilians-University MunichMunich, Germany; 2 Department of Animal Science, Faculty of Agriculture, University of ZagrebZagreb, Croatia; 3 Institute of Animal Sciences, The Faculty of Agriculture, The University of SarajevoSarajevo, Bosnia and Herzegovina; 4 Department of Livestock Science, Biotechnical Faculty, University of MontenegroPodgorica, Montenegro; 5 Department for Genetic Resources, Ministry of Agriculture, Forestry and Water ManagementBelgrade, Serbia; 6 Department of Animal Science, Faculty of Agriculture, University of PrishtinaLidhja e Pejes, Prishtina, Kosovo-UNMIK; 7 Bitola Faculty of Biotechnical Sciences, University “St. Kliment Ohridski”Bitola, Macedonia; 8 National Coordinator of FAnGRRr.“Abdyl Frasheri” Nd.5 H.29 Ap.6, Tirana, Albania; 9 SAVE Foundation, Project OfficeSt.Gallen, Switzerland; 10 Institute of Animal Husbandry and Breeding, University HohenheimStuttgart, Germany

**Keywords:** Cattle, conservation genetics, genetic diversity, inbreeding, metapopulation, sustainable breeding

## Abstract

We estimated neutral diversity of 21 European cattle breeds with 105 microsatellites. Nine of them resembled unselected Balkan Buša strains with diffuse breeding barriers and the 12 others were strongly differentiated, isolated breeds. Because of the impact of neutral genetic diversity on long-term population adaptive capacity, we discuss the long-term outcome of different conservation priorities in a subdivided metapopulation of the investigated cattle breeds. The optimal contribution to a pool of total genetic diversity allocated more than 95% of long-term relevant neutral diversity to virtually unselected strains of the Balkan Buša, while the maximization of total variance preferred inbred breeds. Current artificial selection methods, such as genomic selection sped up and a recovery of underestimated traits becomes quickly impossible. We emphasize that currently neutral and even deleterious alleles might be required for future genotypes in sustainable and efficient livestock breeding and production systems of a 21st century. We provide cumulative evidences that long-term survival relies on genetic complexity and complexity relies on allelic diversity. Our results suggest that virtually unselected, nonuniform strains harbor a crucial proportion of neutral diversity and should be conserved with high global priority. As one example, we suggest a cooperative maintenance of the nondifferentiated, highly fragmented, and fast vanishing metapopulation of Balkan Buša.

## Introduction

Population genetic diversity is the basis of evolutionary potential of species to respond to environmental changes (e.g., Markert et al. 2010). In population genetics, gene diversity is usually equated with heterozygosity (Jost 2008). Here, we use it as an umbrella term for the degree of genetic variation including allelic diversity. An increasing number of wild species and domestic breeds require human intervention to guarantee their survival, and genetic diversity is a crucial point in the choice of conservation priorities (Frankham et al. 2002; Toro and Caballero 2005; Markert et al. 2010). Wild species including wild progenitors and traditional unselected strains of farm animals have evolved as homeostatic organisms that can respond to diverse environmental challenges in different ways (compare Badyaev 2011 and Stern and Orgogozo 2009). Biological traits, particularly relevant to long-term survival, have evolved by noise-tolerant evolution based on phenotypes, not genotypes (e.g., Weiss and Fullerton 2000). On the other hand, in intensively managed domesticated species the selection process is usually a conscious decision with a well-defined and rather short-term goal (e.g., Andersson and Georges 2004). Artificial selection relied on phenotypes, too, but only a few characteristic traits were very important during the foundation period of breeds. Many of intensively selected and managed breeds are no longer populations of homeostatic organisms but have to function in intensive systems that are disconnected from natural diverse challenges, and thus from gradual adaptation to a changing environment. In farm animals and particularly in cattle, the artificial selection pressure has increased very rapidly since the advent of quantitative genetic methods and artificial reproductive techniques, namely artificial insemination, embryo transfer, and use of sexed semen (Powell et al. 2003; Sørensen et al. 2011). This led to impressive ratios between a low effective population size and a huge census number in economical successful breeds (Taberlet et al. 2008). Reduced fitness and accumulation of hereditary and “production diseases” were a result (Dobson et al. 2007; O'Neill et al. 2010), and through a critical low effective population size, the entering of an extinction vortex is not beyond the actual threats of domestic species (Taberlet et al. 2008). In a most recent trend, cattle breeders have started to use genomic selection that relies on genotype and only post hoc tests of phenotypes (Hayes et al. 2009). Because of the intensity of artificial selection, a recovery of underestimated traits may become quickly impossible. Emerging diseases, climate changes, and changes in the nutritional needs of the global human community are unforeseeable. Thus, overall genetic resources defined by adaptive and neutral diversity must be maintained in order to conserve the potential to react to the future challenges, and it has been well recognized that local autochthonous breeds represent a genetic resource in lack of or in addition to a wild ancestor (Taberlet et al. 2008; Medugorac et al. 2009).

This study explores the development of diversity caused by different intensities of selection for production traits in farm animals. Furthermore, we demonstrate the evolutionary genetic consequences of current breeding strategies in taurine cattle in dependence on the applied intensity and direction of selection. Breeds that are either highly selected and hosted in favorable environments (environmental pressures are managed through interventional husbandry strategies) or virtually unselected and hosted in challenging environments (pressure of more “natural” environments) are regarded. We discuss different conservation methods that are compatible with the existing population genetics theory but lead to diametric results. First, we look at the maximization of the total genetic variance of a hypothetical trait (*MVT*; Bennewitz and Meuwissen 2005), which implicitly gives two times more weight to between- than within-population diversity. Our second method is the optimal contribution to a pool of total genetic diversity (*GD_POOL_*; Caballero and Toro 2002) that gives equal weights to within- and between-population diversity. The third method is the maximization of the observed number of neutral alleles within a pool of protected subpopulations or breeds (*NC;*Bonin et al. 2007). By conserving the maximal number of complementary neutral alleles, *NC* maximizes the allelic richness (AR) of the protected pool and thus, according to the discussion in Caballero and Toro (2002), should be equivalent to the *GD_POOL_* approach. Different weighting of within- and between-subpopulation diversity results in diametric conservation concepts (Bennewitz and Meuwissen 2005). One prefers short-term response (*MVT*) coupled with inbreeding depression and direct dependence on expected heterozygosity (*H_E_*) (Caballero et al. 2010), and others (*GD_POOL_* or *NC*) prefer subpopulations with high AR (even at comparable heterozygosity; Caballero et al. 2010) coupled with long-term selection and the potential for adaptation. We discuss consequences of applying different concepts, provide some derivations and perspectives on conservation strategies in a subdivided metapopulation and discuss how to contribute to the sustainable livestock systems in highly variable challenging environments.

## Materials and Methods

### Breeds and strains

We used the data of 21 European cattle subpopulations. These subpopulations are characterized as breeds or strains depending on their breeding monitoring level and administrative isolation. While animals of commercial breeds fulfill some phenotypic standards defined by breeding organizations and are subjected to recording (pedigree and phenotypes), assortative mating, and administrative isolation, local strains are more diffuse domestic subpopulations. In general, local strains are formed by many more or less fragmented subpopulations without organized recording, selection, strict breeding isolation, and monitoring by breeding associations (see Cinkulov et al. 2008; Ramljak et al. 2011).

Another subdivision of the domesticated cattle species follows the geographic distribution into some cosmopolitan and many local subpopulations. Commercial cosmopolitan breeds are isolated from local subpopulations by their administration. Local subpopulations can be divided into two groups: (1) local breeds, that have been managed in the past and are well differentiated but have lost economic importance and remain in small census numbers with a small effective population size and (2) local strains, that have never been intensively managed or differentiated, whose effective population size is still large but whose census number is dramatically decreasing.

The core sample set of this study consists of nine virtually unselected autochthonous local strains of the Balkan with more diffuse barriers (Fig. S1) and is embedded within 12 European reference breeds under different selective monitoring. These nine local strains are summarized as Balkan Buša cattle. Buša (in Albanian written as Busha) is a collective term for small and robust cattle, their withers’ height at around 100 cm. Archaeological findings demonstrated that the Bronze Age cattle kept their size (100–115 cm) until the Late Middle Ages (Davis 1995, p. 178), when an increase in size in most European cattle started to be noticed. The Buša cattle kept its small size since then, thus, it represents a valuable relict of a long adaptation and selection that probably focused on trait values concerning reproduction and modest production in harsh environments. Due to the small size, these Buša strains were not well suitable for work and have been bred for dairy and beef over almost the entire Balkan and parts of Turkey until now. A common and important feature of all Buša strains is that they show high fitness (high fertility and long life history) in challenging environments with low managerial input, that is, low-quality feeds, poor housing, and infrequent use of drugs to prevent or cure disease. It is very common, that cows give yearly birth to altogether 12–16 calves during their life. It has been shown that the total lifetime yield for Buša is higher than for commercial cows, especially if the challenging environment and the 2–3 times lower body weight is taken into account (e.g., Šmalcelj 1956). Three Buša strains have not previously been analyzed and are described in detail in Figures S2, S3, and S4: Bosnian-Herzegovinian Buša (BHB), Montenegrin Buša (MNB), and Prespa Cattle (PRB). Eighteen breeds are previously described in Medugorac et al. (2009) and Ramljak et al. (2011). Six of them belong to the Buša group, Macedonian Buša (MBU), Illyrian Mountain Buša (IMB), Illyrian Lowland Buša (ILB), Red Metohian Buša (RMB), Gray Gacko Buša (GGB), and Croatian Buša (HRB). Figure S1 shows a map with the origin of the Buša samples. The podolian breeds from the Mediterranian and Pannonian region are Istrian Cattle (HRI) and Slavonian Syrmian Podolian Cattle (HRP), respectively. The dual-purpose breeds from the Alpine region are Tyrolean Grauvieh (TGV), Original Braunvieh (OBV), Murnau-Werdenfelser (MWF), Austrian Murbodner (AMB), Franken Gelbvieh (FGV), Fleckvieh (FV), and Tarentaise (TAR). Three northwestern highly specialized breeds are Red Holstein (RH), Blanc-Bleu Belge (BBB), and Galloway (GLW). Two Alpine breeds, TGV and OBV, have mostly been used for upgrading of Balkan cattle during the past. In Table 1, all 21 breeds with their origin, purpose of the selection, and sample size are listed.

**Table 1:** Breeds, codes, and geographic origin. In addition, purpose of the selection and the environmental conditions in which the breed is usually kept, and number of genotyped samples (*N*) are listed. Three additional breeds, that have not been previously described, are in bold. The core sample set of nine Buša subpopulations is marked gray

Breed	Code	Origin	Purpose of the selection^1^	Environments	*N*
Macedonian Buša	MBU	Macedonia	Dairy-beef (work)^2^	Challenging	31
**Prespa Cattle**	**PRB**	**Albania**	**Dairy-beef (work)**	**Challenging**	**50**
Illyrian Mountain Buša	IMB	Albania	Dairy-beef (work)	Challenging	45
Illyrian Lowland Buša	ILB	Albania	Dairy-beef (work)	Challenging	29
Red Metohian Buša	RMB	Kosovo - UNMIK	Dairy-beef (work)	Challenging	44
**Montenegrin Buša**	**MNB**	**Montenegro**	**Dairy-beef (work)**	**Challenging**	**43**
**Bosnian-Herzegovinian Buša**	**BHB**	**Bosnia-Herzegovina**	**Dairy-beef (work)**	**Challenging**	**49**
Gray Gacko Buša	GGB	Bosnia-Herzegovina	Dairy-beef (work)	Challenging	41
Croatian Buša	HRB	Croatia	Dairy-beef (work)	Challenging	51
Slavonian Syrmian Podolian Cattle	HRP	Croatia	Work-beef	Favourable	51
Istrian Cattle	HRI	Croatia	Work-beef (dairy)	Favourable	51
Tyrolean Grauvieh	TGV	Austria	Dairy-beef	Favourable	48
Original Braunvieh	OBV	Germany	Dairy-beef	Favourable	46
Murnau-Werdenfelser	MWF	Germany	Dairy-beef	Favourable	53
Austrian Murbodner	AMB	Austria	Dairy-beef	Favourable	47
Franken Gelbvieh	FGV	Germany	Dairy-beef	Favourable	48
Fleckvieh	FV	Germany	Dairy-beef	Favourable	55
Tarentaise	TAR	France	Dairy-beef	Favourable	39
Red Holstein	RH	Germany	Dairy	Favourable	50
Blanc-Bleu Belge	BBB	Belgium	Beef	Favourable	47
Galloway	GLW	Germany (Scotland)	Beef	Favourable	47

1 Sporadic or more accessory purpose are put in parentheses.

2 Multi-purpose selection is directly associated with lower selection intensity for each of the respective trait.

### DNA extraction and microsatellite analyses

A total of 105 microsatellites was used and all 965 samples were genotyped twice in two independent courses. The standard methods of genomic DNA extraction, PCR amplification, microsatellite genotyping, and exclusion of 12 outliers (Table S1) followed the protocols already described for the other 18 breeds (Medugorac et al. 2009; Ramljak et al. 2011).

### Genetic variability

Estimates of genetic variability, observed heterozygosity (*H_O_*) and (*H_E_* (Nei 1987), *AR* (El Mousadik and Petit 1996), and *F*-statistics (Weir and Cockerham 1984) for each locus including population pairwise *G_ST_*, were determined using *fstat* v.2.9.3. (Goudet 2001). The estimator of the true population differentiation *D_EST_*(Jost 2008) was predicted as harmonic mean of *D* values across loci. Here, we used our own application (IM not published data) that implements the approach described by Crawford (2010) but is applicable to larger datasets. Fisher's exact test was used to determine the deviation from Hardy–Weinberg equilibrium (HWE) using *genepop* v.4.0 software package (Raymond and Rousset 1995). Unbiased estimates of exact *P*-values were obtained by the Markov Chain Monte Carlo algorithm. The alleles were classified in three levels, according to their frequency; common alleles (observed in all subpopulations), private alleles (*pA*) (alleles observed in one subpopulation), and rare alleles (*rA*) that are nonprivate alleles with an arbitrary chosen frequency less than 0.01 over the whole population. This criterion mostly implies a frequency of <5% in the respective subpopulation according to the total sample size of 965, an average sample size of 46 animals, and a distribution of *rA* (present in <20 gametes) mostly over two to three subpopulations.

### Effective population size based on LD (*Ne_ld_*)

We estimated *Ne_ld_* on the basis of the marker–marker *LD* (χ^2^_*df*_) by weighted least squares regression, which takes into account the heterogeneity of the *LD* variances (Zhao et al. 2005). This was done for all neutral marker pairs of chromosomes 1, 2, 3, and 6 (here highest marker density, 21, 7, 9, and 18, respectively) for the three newly analyzed breeds with the same procedure as described in Medugorac et al. (2009). *Ne_ld_* does not directly represent the true effective population size (*Ne*) but it is well related (Zhao et al. 2005). Therefore, *Ne_ld_* can be understood as an estimator of haplotype diversity as well as a relative value of *Ne*.

### Assignment analyses and clustering analysis

To infer overall relationships between the breeds, *D_A_* distances (Nei et al. 1983) were calculated. A neighbor-joining consensus tree (1000 bootstrap replicates) was constructed with the program *phylip* (Felsenstein 1993). The tree and the neighbor network were plotted with the program *splitstree4* (Huson and Bryant 2006).

For assignment and structure analyses, the nine Buša strains and the two Alpine breeds TGV and OBV were used, because they have been used intensively to upgrade the Balkan cattle population during the past (especially GGB and HRB; for more detail see Medugorac et al. 2009 and Ramljak et al. 2011, respectively). An assignment test was done with the program *geneclass* (Piry et al. 2004) based on multilocus genotypes and the method first described by Paetkau et al. (1995), 1000 individuals were simulated. *structure 2.2*(Pritchard et al. 2000; Falush et al. 2003) was implemented to determine the most likely number of clusters (*K*) in the dataset, independent of breed affiliation. Ten independent runs of *K* = 1 to 11 were carried out, burn-in period 50,000 and 200,000 replicates. To determine the most likely hierarchical structure, we used the log probability of data *LnP(D)* and estimated the delta *K* (*ΔK*) statistics as explained in Evanno et al. (2005). The *structure* results were plotted with *distruct* (Rosenberg 2004).

### Strategies for conservation in subdivided populations: neutral allelic diversity with principle of complementarity (*NC*)

In total, 105 microsatellites were genotyped in 21 cattle subpopulations. For phylogenetic and conservation studies, 93 neutral markers were chosen (see Medugorac et al. 2009 and Ramljak et al. 2011). Therefore, all observed alleles at these 93 markers are considered as neutral alleles. Similar as in Bonin et al. (2007), *NC* is defined as the proportion of neutral alleles conserved by a pool of 1 to *n* subpopulations. This method maximizes the observed number of neutral alleles within a pool of *x* (*x* = 1 to *n*) protected subpopulations or breeds.

### Minimizing coancestry (kinship) in a subdivided metapopulation

Eding et al. (2002) developed a method to estimate conservation priorities within a sample set of breeds. This method aims to define a core set of prioritized breeds with a minimized mean kinship based on an estimate of the relative contributions of the breeds under consideration. An analogue of the Eding's core set method was used by Caballero and Toro (2002), the average coancestry between and within subpopulations for the description of genetic diversity. They also demonstrated that minimization of coancestry in a subdivided population is equivalent to maximization of effective population size in a pool of protected subpopulations. In order to estimate the optimal contribution from each subpopulation to a pool of maximal gene diversity (*c_GDpool_*), the methods described by Caballero and Toro (2002) and implemented in the software *metapop 1.0.1* (Pérez–Figueroa et al. 2009) were used. This method implicitly gives equal weights (λ) to within- and between-population diversity (i.e., λ = 1; see Meuwissen 2009). It is expected that the maximization of genetic diversity will lead to maximum AR in long-term conservation programs (Caballero and Toro 2002). Therefore, also the *NC* approach above implicates λ = 1 (see Meuwissen 2009).

### Core set of breeds with maximized total genetic variance (*MVT*)

The *MVT* diversity of the core set is calculated as described in Bennewitz and Meuwissen (2005). The relative breed contributions (*c_MVT_*) reflect the importance of the breeds for the actual diversity defined by the total genetic variance of a hypothetical quantitative trait, which implicitly gives two times more weight to between- than within-population diversity (λ = 0.5; Meuwissen 2009). The *MVT* core set favors breeds with a high within-breed kinship that are not related to other breeds. Following this, the *MVT* core set method suggests conserving breeds that show a large difference in the respective population mean of a hypothetical quantitative trait (Bennewitz and Meuwissen 2005).

### Simulation of metapopulations and depleted bottleneck subpopulations

To corroborate a decision-making process and to illustrate different outcomes of distinct strategies for conservation in subdivided populations, we added four simulated populations to our samples. These synthetic populations were derived from the real sample data. This relatively simple procedure was carried out with an own *fortran* application (IM not published data). First, the nine Buša subpopulations were divided into two metapopulations (MetaB1, MetaB2) each containing one-half of randomly chosen individuals. This subdivision was performed within strains, therefore both MetaB1 and MetaB2 contain one random half of each of the nine Buša subpopulations. Second, the most diverse Buša subpopulation, RMB, was used to derive the simulated subpopulation of small effective population size. In successive steps, RMB was downsized and then reproduced by random mating. We used a random mating of 44 RMB animals to form the next generation of 42 animals. After subsequent 13 non-overlapping generations, a final size of 20 animals was reached. These were reproduced at constant population size for the next 10 generations again by random mating. This led to the depleted population RMBD. Third, following the procedure for RMBD, the most diverse Alpine breed, FV, was also downsized to 20 animals and simulated as the depleted subpopulation FVD. For both RMBD and FVD, the random mating allowed that one random animal could have several offspring while another could have none.

Finally, we considered a set of 16 subpopulations including (1) all 12 non-Buša breeds without any changes, (2) two by simulation depleted subpopulations, and (3) two metapopulations each including the half random chosen Buša animals. This set of 16 subpopulations was analyzed by all three conservation strategies.

## Results

### Genetic diversity

Table S1 shows all 105 loci, their relative position in the cattle genome, the observed number of alleles (*nA*), and the *H_E_* and *H_O_* over all 965 individual animals. The genetic variability of BHB, MNB, and PRB with 0.724, 0.723, and 0.728, respectively, is within the Buša range of 0.723–0.747. Table 2 shows the observed total number of alleles (*tA*), *pA* and *rA, AR, H_E_, H_O_*, and the *F_IS_* statistic estimated for 21 breeds for 93 neutral loci. According to previous results (Ramljak et al. 2011), 12 loci with decisive evidence for being under selection or in HW disequilibrium were consequently excluded from the further analyses requiring neutrality (Table S1). Table S2 shows population pairwise *G_ST_* and *D_EST_* values, and in Figure S5 the tree and the network of the distance matrix are found.

**Table 2:** Summary statistics of neutral genetic diversity of 21 cattle breeds. Total number of alleles (*tA*), number of private alleles (*pA*), number of rare alleles (*rA*) and allelic richness (*AR*), unbiased expected heterozygosity (*H_E_*), observed heterozygosity (*H_O_*), fixation index (*F_IS_*). The last two rows show mean value and standard deviation

Breed	*tA*	*pA*	*rA*	*AR*	*H_E_*	*H_O_*	*F_IS_*
MBU	715	5	98	7.42	0.744	0.694	0.015
PRB	736	9	86	6.98	0.728	0.690	0.025
IMB	760	8	102	7.25	0.726	0.693	0.022
ILB	704	7	69	7.33	0.726	0.667	0.052
RMB	815	16	133	7.81	0.747	0.721	0.025
MNB	763	5	100	7.29	0.723	0.683	0.040
BHB	731	14	105	6.89	0.724	0.654	0.047
GGB	708	3	73	6.90	0.717	0.667	0.049
HRB	792	10	122	7.39	0.730	0.648	0.089
HRP	451	3	18	4.46	0.583	0.593	–0.026
HRI	634	4	48	6.01	0.677	0.635	0.036
TGV	554	4	31	5.44	0.663	0.652	–0.007
OBV	605	3	47	5.88	0.678	0.660	–0.009
MWF	529	6	29	5.22	0.661	0.657	–0.016
AMB	574	3	38	5.67	0.665	0.661	–0.031
FGV	562	3	37	5.54	0.643	0.625	–0.006
FV	629	2	51	5.92	0.667	0.660	–0.002
TAR	544	0	20	5.50	0.654	0.630	0.011
RH	577	6	32	5.61	0.663	0.642	0.017
BBB	584	3	39	5.72	0.661	0.619	0.026
GLW	511	3	21	5.00	0.616	0.565	0.069
Mean	641	5.6	61.9	6.25	0.686	0.653	0.020
SD	101	3.9	35.3	0.94	0.043	0.035	0.030

### Assignment test and structure results

**Figure 1:**
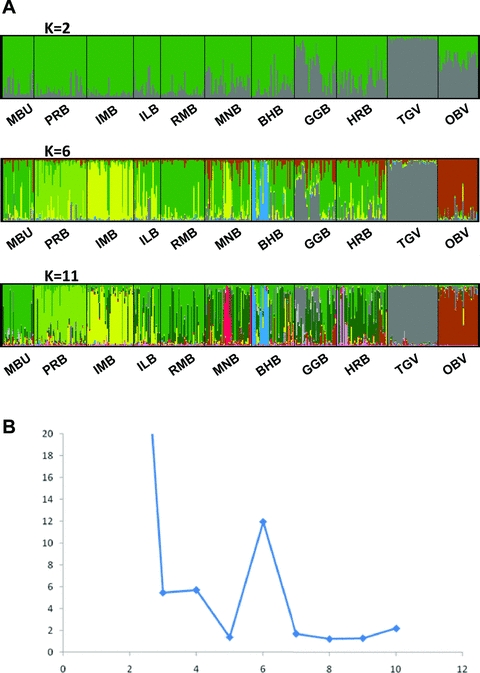
The structure results of the nine Buša strains and two Alpine breeds, TGV and OBV are shown for *K* = 2, *K* = 6, and *K* = 11. TGV and OBV were found in distinct clusters, also PRB and, with increasing *K*, IMB. The remaining seven Buša strains showed a more diffuse clustering with single groups of individuals within a local strain being concise clusters rather than the whole subpopulation (A). Maximum delta *K* values are shown (B).

The results of the assignment test and *structure* are presented for the nine Buša subpopulations and the two Alpine breeds (TGV and OBV) that have been used intensively for upgrading of cattle populations in the Buša sampling region during the past. The assignment test directed 185 (44%) of the 420 individuals to their respective populations. The structure results of the nine Buša strains and two Alpine breeds, TGV and OBV, and the *ΔK* values are shown in Figure 1A and 1B for *K* = 2, *K* = 6, and *K* = 11 with highest *ΔK* values. TGV and OBV were found in distinct clusters, also PRB and, with increasing *K*, IMB. The remaining seven Buša strains showed a more diffuse clustering with single groups of individuals within a local strain being concise clusters rather than the whole subpopulation.

### Strategies for conservation in subdivided populations

In Table 3, the range of breeds is shown, that is prioritized by three different conservation strategies *NC, c_MVT_*, and *c_GDpool_*. Apparently, *c_MVT_* prioritizes inbred breeds with a known recent bottleneck such as HRP, GLW, MWF, and TGV. Six prioritized breeds with *c_MVT_*≥ 10% (HRP, TGV, MWF, GLW, TAR, and HRI) have *AR*, numbers of observed total, private, and rare alleles (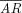 = 5.27, 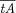 = 537, 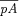 = 3.3, 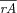 = 27.8) well below average (Table 2) and are effectively small (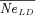 = 201.9; Table 3). The *H_O_* and *H_E_* of these six breeds is also below average of all 21 breeds. In contrast, *c_GDpool_* prioritizes the Buša strains and shows that 95% of the total genetic variance could be solely preserved with them. Five of those show 10% and more (e.g., RMB 28%) contribution to the total genetic pool and account for 94.5%. These five populations have a significantly higher *AR*, higher numbers of observed total, private, and rare alleles (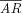 = 7.27, 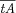 = 751, 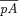 = 10.4, 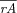 = 104.8; Table 2), are effectively larger (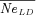 = 371.5; Table 3) and show *H_E_* and *H_O_* above average of all 21 breeds. The relatively simple conservation criterion *NC* also prioritizes the effectively large Buša strains (Table 3). According to the *NC* criterion, seven highly diverse and effectively large (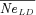 = 405.9) Buša subpopulations conserve 94% of total allele diversity with average *AR* of 7.30.

**Table 3:** Effective population size (*Ne*) and conservation priorities. *Ne*_LD_ is estimated on the basis of the marker–marker LD. Neutral diversity with principle of complementarity, presented by the cumulative number of neutral alleles (*NC*) when the first *x* subpopulations are combined, NC is the proportion of neutral alleles that would be conserved with the respective *x* breeds. Conservation priority estimated by maximization of total variability (***c****_MVT_*) in comparison with contribution to a maximized genetic pool (***c****_GDpool_*). *Ne*_LD_ is estimated based on the original set of 21 breeds or strains. All three conservation priorities are estimated for original and modified dataset. The modified dataset includes two metapopulations (MetaB1 and MetaB2), two depleted populations (RMBD and FVD), and 12 non-Buša breeds. MetaB1 and MetaB2 include random half of each of the nine Buša strains

Breed	Original dataset					Modified dataset			
		
	*Ne_LD_*	*NC*	*x*	*c_MVT_*	*c_GDpool_*	*NC*	*x*	*c_MVT_*	*c_GDpool_*
MBU	302.6	0.889	4	0	25.8	—	—	1—	—
PRB	393.8	0.913	5	3.33	19.5	—	—	—	—
IMB	358.4	0.957	9	8.36	10.6	—	—	—	—
ILB	306.4	0.940	7	0	0	—	—	—	—
RMB	521.9	0.710	1	0	28.7	—	—	—	—
MNB	474.6	0.927	6	0	0	—	—	—	—
BHB	280.8	0.857	3	0	9.9	—	—	—	—
GGB	405.2	0.996	18	0	0	—	—	—	—
HRB	560.9	0.806	2	0	0.7	—	—	—	—
HRP	117.0	0.988	15	19.20	0	0.979	6	20.02	1.1
HRI	255.1	0.976	12	9.90	4.7	0.990	9	8.03	12.1
TGV	199.6	0.984	14	10.98	0	0.987	8	4.82	1.2
OBV	287.2	0.990	16	0	0	0.993	10	0	0
MWF	149.2	0.949	8	10.10	0	0.969	4	9.11	0
AMB	200.8	0.965	10	0	0	0.962	3	0	0
FGV	274.6	0.998	19	3.33	0	0.998	12	0	0
FV	409.9	1.000	20	0.02	0	1.000	13	0	0
TAR	249.7	1.000	21	9.74	0	1.000	13	0.96	1.1
RH	272.9	0.971	11	7.91	0	0.975	5	0	0.4
BBB	231.8	0.981	13	7.13	0	0.983	7	1.29	2.6
GLW	241.2	0.993	17	10.01	0	0.996	11	13.87	0
FVD	—	—	—	—	—	1.000	13	21.73	0
RMBD	—	—	—	—	—	1.000	13	20.15	3.5
MetaB1	—	—	—	—	—	0.953	2	0	36.6
MetaB2	—	—	—	—	—	0.899	1	0	42.4

1 Not applicable

The results of the simulated populations follow the trend outlined above. Both FV and RMB do not appear in the ranking of the *c_MVT_* approach. FV resembles the most diverse and well-monitored Alpine breed with a large effective population size and highest *tA, rA*, and *AR* between all Alpine and northwestern breeds. RMB is highly diverse with a large effective population size, but is a traditional Buša strain without strict monitoring. By mimicking a bottleneck and a loss of neutral genetic diversity by strong drift, these two depleted simulated breeds (FVD and RMBD) appear on top of the conservation priority of *c_MVT_*. HRP is a Croatian strain of a formerly large podolian cattle population of the Pannonian plain. It is well documented (Ramljak et al. 2011) that this breed passed a very strong genetic bottleneck but was not subjected to strong directed artificial selection. This is the natural counterpart to the simulated breeds RMBD and FVD and shows virtually the same *c_MVT_*. In contrast, the *c_GDpool_* (Metapop approach) did not prioritize either FV or FVD. In addition, RMB as a highly diverse subpopulation was listed highest with *c_GDpool_*. After the simulated bottleneck and substantially loss of neutral diversity (*tA* = 286, i.e., 35% of original allele diversity, *rA* = 4 [3%], *pA* = 0 [0%]), only a limited (3.5%) *c_GDpool_* contribution was estimated for RMBD. Remarkably, this contribution is still exceeding the contribution of well-managed modern breeds (0.0–2.6%).

MetaB1 and MetaB2 are a combination of individual animals of highly diverse strains into two metapopulations. *NC* gave highest priority for these two metapopulations but lowest for two artificially depleted breeds (FVD and RMBD, Table 3, modified dataset).The *c_MVT_* approach gave some priority to two Buša strains (PRB 3.33% and ILB 8.36%) before the random fusion but none thereafter. By minimizing coancestry in a subdivided metapopulation, we estimated a cumulative *c_GDpool_* = 95.2% of nine Buša strains (Table 3) before and 79% after the random fusion.

## Discussion

The applied methods for conservation in subdivided populations mainly differed for their relative weights (λ) given to within- versus between-population diversity. These methods representing different conservation concepts have been compared (Bennewitz and Meuwissen 2005), and it is well known that they will produce different and sometimes opposite conservation priorities (Meuwissen 2009; Toro et al. 2009). Therefore, this study does not aim to compare methods on field data—this has to be done on data with known parameter, that is, simulated data—but to discuss long-term consequences of applying different concepts.

As discussed by Caballero and Toro (2002), their approach corresponds with maximization of effective population size being inversely related to inbreeding. The *Ne_LD_* estimator of effective population size is 1.84 times larger in five *c_GDpool_*-prioritized Buša strains than in six *c_MVT_*-prioritized breeds. The *MVT* core set approach aims to conserve breeds that show comparatively large differences in the respective population mean of a hypothetical quantitative trait. This seems to make the *MVT* core set method attractive, because the efficiency of upgrading a breed by introducing alleles from another breed is a function of the difference between the respective population means (Bennewitz and Meuwissen 2005). Thereby, homogeneous populations that have lost a substantial proportion of within population diversity by random drift (e.g., HRP) or by strict selection (e.g., GLW) are prioritized. This characteristic of the *c_MVT_* approach is known and intentional (Bennewitz and Meuwissen 2005), because the method aims to maximize the speed of achieving selection response for a putative changed breeding objective by use of genetically consolidated and inbred breeds. Furthermore, the concern is that an overemphasis on within-breed variation (λ = 1) will favor the largest breeds, which are commercially more valuable and, therefore, less endangered, but an overemphasis on between-breed variation (λ = 0) may result in favoring inbred populations even though they might not contain specific interesting alleles (Toro et al. 2009). Thus, some compromise (i.e., λ = 0.5) should be attempted (Meuwissen 2009). Our results do not reflect this argument. The compromise (*c_MVT_*) still overemphasizes on between-breed variation and gives the by far highest priority to a population with the lowest diversity (HRP; *AR* = 4.46; *H_O_* = 0.583) that is not unified by systematic selection but simply by chance due to a strong bottleneck (Ramljak et al. 2011). Contrary, giving equal weights to within- and between-population diversity (*c_GDpool_*) does not favor the commercially breeds with large census sizes (but mostly effectively small) but the virtually unselected strains of the Buša metapopulation with a large effective population size. Four synthetic populations derived from the sample data corroborate this. Simple depletion by chance of the Alpine breed (FVD; effectively large and with large census number) or the most diverse Buša strain (RNBD) led to *c_MVT_* values comparable with HRP (21.7, 20.1, and 20.0, respectively, Table 3). Therefore, using a modified dataset (Table 3), the *c_MVT_* approach would clearly favor three inbred populations even though they do not contain specific interesting alleles, while the *c_GDpool_* approach would favor the high neutral allelic diversity of effectively large populations, whether as metapopulation or not. The reason is that all applied methods do not differentiate between subpopulations uniformed by strong adaptive or artificial selection and subpopulations depleted by chance in a strong bottleneck. This challenges the primary assumed advantage of the MVT core set that should enable a faster reaction on putative changed conditions compared to methods that emphasis more on within-breed variation. If higher priority is given to already selected, consolidated, and inbred breeds with regard to an efficient future upgrading, the assumption of similarities between present and future breeding goals is implicit. However, this is questionable in the expectance of a severe change in environmental conditions and nutritional habits of the human community.

### Neutral genetic diversity reflects fitness of domestic animals

Livestock production in the 21st century will be characterized by systems where both market (food and fiber output) and non-market (social, environmental, and climate change) issues are given consideration. Thus, livestock producers will maintain their drive for maximization of output but with diminished reliance on industrialization and therapeutic measures to alleviate environmental stress and rely more on the genetic make-up of their animals to combat stress from heat, parasites, pathogens, and poor nutrition (O'Neill et al. 2010). Fitness is understood as an individual's ability to grow and reproduce viable offspring. It will be adversely affected, if the animal is unable to mount an appropriate response to the environmental stressors (either biotic or abiotic factors) via means of its physiology and/or behavior. The degree of sensitivity to these stressors, meaning the ability to maintain homeostasis (self-regulation of the individual or on the higher level of the population), is thus an aspect of fitness (Falconer and Mackay 1996). Mounting evidence indicates that biodiversity loss frequently increases disease transmission between hosts on population level and in the first place enables disease manifestation on an individual level (Keesing et al. 2010). There is an urgent demand for unknown alleles and phenotypes of future fitness to be mobilized from the pool of current neutral diversity of rather non-uniform populations. Present-day variation in livestock associated with economically important traits may have been selectively neutral in the past. At least, the high productive variants would not have been beneficial or adaptive in most of past challenging environments. Many of today's important economic traits were not monitored until a century or less ago. Variation in a gene associated with such a trait could thus have evolved neutrally, unless there were some early onset pleiotropic effects. The most important economic trait in the cosmopolitan cattle breed Holstein-Frisian is milk protein yield, which started to be systematically recorded after the 1970s. The Blanc-Bleu Belge (BBB) cattle is today understood as the most obvious phenotypic opponent of Holstein-Frisian dairy cattle. Both breeds are cosmopolitans, have the same geographical origin, and the double-muscled phenotype, now typical for BBB, segregated in both breeds at the beginning of the 20th century. The very recent divergent selection resulted in enormous morphological differentiation between Holstein and BBB (Fig. S6). On the other hand, as expected, the neutral genetic differentiation between RH and BBB (both *G_ST_* and *D_EST_*; Table S2) does not reflect this short-term phenotypic differentiation essentially based on some major genes (Medugorac et al. 2009; Fig. S5).

The cattle breeds chosen for this study present a wide spectrum of productivity in current farm animals. On the one hand, there are virtually unselected Buša strains (e.g. Figs. S2, S3, and S4) with high fitness (high fertility and long life history) in a challenging environment, but with relatively low annual output; on the other side of the spectrum are breeds with low fitness (e.g., Dobson et al. 2007) and inbreeding depression (e.g., Carrillo and Siewerdt 2010) but high annual output in a favorable environment where environmental pressures are managed through intervention strategies such as providing high-quality feeds, artificial housing, or disease management. Our results clearly demonstrate high neutral diversity in the first group and suggest their potential for economically and environmentally sustainable agriculture.

### Parallels of selection response limits and evolutionary limits

The applied methods for conservation priorities led to diametric results but later we will favor the results of the *c_GDpool_* concept (Caballero and Toro 2002; Eding et al. 2002). To underpin this decision, which appreciates neutral diversity as the basis of long-term conservation, we describe the long-term survival of a subdivided population or species by classical quantitative genetic theory and draw a parallel to theoretical selection limits (Falconer and Mackay 1996, p. 218). Both depend on initial genetic variation (σ_*A*_) and total possible response (*R_T_*). The long-term survival relies on balance between the best possible current adaptation (i.e., σ_*A*_→*Min*) and a widest open evolutionary window for future developments (i.e., *R_T_*→*Max*). Similar as demonstrated for selection response (Falconer and Mackay 1996, p. 219), simultaneous maximizing of *R_T_* and minimizing of σ_*A*_ is achieved by maximizing the term *R_T_*/σ_*A*_. By use of simplified models it could be shown that maximal long-term survival is achieved at a maximal number of segregating genes *R_T_*/σ_*A*_→ 8*n*→*Max*. This can be interpreted as follows: the evolution maximizes the probability of the long-term survival by consistent increase of the number of polymorphic genes affecting survival, that is, by consistent increase of complexity, 8*n*→*Max* = ∞. Hill and Rasbash examined various more realistic assumptions about the distribution of gene effects and gene frequencies (1986a) including recurrent mutation (1986b). These studies confirmed the primary function of the number of segregating loci and underline the important role that the effective population size can play in response to long-term selection objectives.

The theoretical terms of selection or evolution limits used above can be easily interpreted in the sense of a long-term conservation strategy. The best possible adaptation to a current environment (i.e., σ_*A*_→*Min*) can be achieved with a lower frequency of currently deleterious or neutral alleles of each single polymorphic locus. But in effectively small populations, the allele with a very low frequency can be often get lost by genetic drift. Likewise, selection can easily drive to fixation of an adaptive locus. Both is directly penalized by the reduction of the evolutionary window (*R_T_*) and consequently decreases the long-term survival probability (*R_T_*/σ_*A*_→*Max*), even if currently not favorable variants are affected by the loss. Therefore, the number of segregating loci (*n*) should be maintained or even increased by recurrent mutations. The maintenance of new and low frequent alleles is more probable in effectively large populations (Hill and Rasbash 1986b). The balance between short- and long-term evolutionary forces is reached at a high number of polymorphic loci affecting survival traits with a lower frequency of currently deleterious or neutral alleles in an effectively large population. Strong selection, that is, picking only few extreme individuals as parents of the next generation, increases short-term but reduces long-term response (Hill and Rasbash 1986b). It also favors alleles with large phenotypic effects, including pleiotropic deleterious effects. Thus, small strongly selected populations may end up selecting far-from-ideal mutations (those with pleiotropic consequences and epistatic effects; Stern and Orgogozo 2009), because potentially superior mutations occur at a lower rate. In addition to that, rare, even advantageous, alleles get lost as a consequence of a low effective population size due to genetic drift. Such loss of *rA* by selection or drift in a subdivided population cannot be restored by any future combination of depleted subpopulations, for example, by crossing, which is the main idea of the *MVT* core set method. This is only possible if a large homeostatic base population would be still available.

### Implications for choice of conservation strategy

The future environmental changes a species will face cannot be predicted, thus, it is not sensible to prioritize a few actual adaptive alleles that may not be the adaptive alleles of the future (Luikart et al. 2003). This is especially true, if the genetic heterogeneity of complex traits is taken into account. Moreover, only a large number of actual neutral alleles, even at a low frequency but present, ensure multiple options for a development in an unforeseeable future environment or—particularly relevant to sustainable agriculture—for new at present unknown nutritional or even other requirements. This long-term aspect of within population diversity is primarily considered by *NC* and *c_GDpool_* (λ = 1), only partly by *c_MVT_* (λ = 0.5) but neglected by the Weitzman (1992) distance approach (λ = 0). The primary loss of diversity within domesticated species is due to the upgrading of local strains by strongly selected breeds instead of admittedly laborious, but required building up of local agricultural infrastructure. Considering this fact and above discussed implication of the similarity of current and future breeding objectives, the conceptual prioritization of highly selected and inbred breeds could in practice rather accelerate the erosion of genetic diversity in livestock species. Instead, the currently neutral or even deleterious alleles, if existent, would need to be activated in future environments. Mankind, as executor breeders and their associations, restricts and even locks the evolutionary window of domesticated species for thousands of animal generations. Some of these are unable to withstand the pressure of more “natural” environments (see O'Neill et al. 2010). The long-term survival of these species or ecosystems or even humankind within ecosystems depends largely upon complexity. This is consistent with recent results of studies (e.g., Keesing et al. 2010; O'Neill et al. 2010) that humans in general and in this context most current farming systems degrade ecosystems to their own peril. Consequently, prioritization of highly diverse and effectively large instead of small and depleted subpopulations seems plausible, sustainable, and in agreement with experimental studies (e.g., Markert et al. 2010). Above parallels of selection and evolution limits can be worked out in a more sophisticated way and possibly used to deduct more appropriate conservation strategies, but this is out of the scope of this present study.

### Conservation management of the subdivided metapopulation of Buša

In practice, following the conclusion reached above, the large neutral diversity of virtually unselected traditional Buša strains should be conserved with a high global priority to ensure sustainable cattle breeding in the future. As we discussed in Ramljak et al. (2011), there is a fast proceeding extinction in most of the here prioritized Buša strains. In well-defined breeds, our set of 93 highly informative markers gave a reliable assignment of individuals to their respective population of 98.8% (Medugorac et al. 2009). In this study, the assignment test was done for nine Buša strains and two Alpine breeds. The reliability dropped to 44%, that is, the Buša strains themselves did not appear as well-defined breeds. Also, in the *structure* results these strains were not very well differentiated except for PRB and IMB and the Alpine breeds, which was to be expected. PRB is found in a geographical isolated position (Fig. S1), migration is hindered. Nei's *D_A_*-distances (Fig. S5) as well as pairwise *G_ST_* and *D_EST_*-values (Table S2) between Buša strains demonstrated their low differentiation. These findings confirm our description of local Buša subpopulations being rather strains with diffuse breeding barriers. In addition, such genetic structures as well as some practical aspects suggest a conservation following the metapopulation approach. The formation of an imaginary metapopulation could overcome inbreeding problems and conserve the maximal within population diversity of Buša strains. It is imaginary because it leaves almost all animals at their original positions and relies on targeted and limited exchange of animals or gametes. This in situ conservation scheme promises more success because the units are kept at different locations. As shown in Table 3, the random distribution of Buša individuals in two arbitrary metapopulations led to a decrease in conservation priority, as between-breed diversity has its own benefit. A controlled gene flow between the different strains could prevent inbreeding without intercrossing all of them. Toro and Caballero (2005) discussed the appropriate gene flow between populations and pointed out that the general compromise of one migrant per generation in conservation could be adapted to the particular situation to account for population structure, census, and effective numbers.

As recording systems have not been established yet, the choice of suitable exchange animals would need to be based on careful genetic information. Above, we pointed out, that even our relatively large marker set could not resolve single individuals to their respective population for most of the Buša strains. Newly developed network-based analysis methods, especially for SNP data, like the Super Paramagnetic Clustering are promising tools to detect fine-resolution population structure without primary ancestry information. Even important founder animals could be highlighted and admixed individuals could be traced back correctly (Neuditschko et al. 2010). Although it is to be expected, that the application to strains with diffuse breeding barriers will challenge this method (I Medugorac, unpubl. results), it is still a promising powerful tool and would be valuable for management decisions about livestock and controlled gene flow between local strains without thorough ancestry information.

Below, we briefly suggest a possible marker-assisted conservation program as an example for a highly diverse and not well-differentiated metapopulation of domesticated animals. First, representative samples of active reproductive animals from each strain should be genotyped by a genome-wide SNP Chip (like the BovineSNP50 BeadChip, Illumina Inc., San Diego, CA, USA). Second, only animals confirmed as not admixed with foreign breeds (i.e., breed not included in metapopulation) should be chosen for exchange. Third, spatially closer neighbors (Fig. S1) are to be preferred as exchange partners. Fourth, in case it is necessary, preferably one animal per year and donor population as a maximum could be introduced in another population. Fifth, the reproduction of the immigrants should be controlled to prevent extinction of host haplotypes and thus decrease of total diversity. Finally, foreign haplotypes, partly introduced into the metapopulation by previous upgrading, can be extinct, if required.

## Conclusions

We investigated cattle breeds in Europe on a wide scale by a relatively large marker set and allocated more than 95% of long-term relevant neutral diversity to virtually unselected strains of cattle in the Balkan area. It is a complex and multifaceted decision-making process to prioritize breeds to participate in a conservation program and other factors than the genetic may need to be considered. However, relating to the latter, we recommend by cumulative evidence to emphasize equally on within- and between-breed variations. We suggested controlling tools for cooperative maintenance of the nondifferentiated but highly fragmented and fast vanishing metapopulation of Balkan Buša. The maintenance of the genetic diversity is a global and long-term task and we appeal for global coordinated efforts to step up the long-term conservation infrastructure. As there are obvious parallels to some other strains of domesticated ruminants, we think that the conservation strategy we suggested for cattle might also be relevant for sheep and goat breeds, and in general for other farm animals or subdivided populations.

## Supporting Information

**Figure S1:** Map of the Balkan area with the origin of the Buša strains.

**Figure S2:** The BHB cow (A) and bull (B) from the Buša rezervat Buhovo (2010).

**Figure S3:** Montenegrin Buša cow (A) and bull (B) from the southeastern coast (Ulcinj 2009).

**Figure S4:** Prespa cow at Liqenas, Albania (A) and a so-called “blue” cow in the Greek Psarades (B).

**Figure S5:** Presentation of Nei's *D_A_*-distance.

**Figure S6:** The phenotypic opponents Holstein Frisian dairy cattle (A) and Blanc-Bleu Belge cattle (B, kindly provided by Alexandre Osio from the Belgian Blue Group).

**Table S1.** Summary information of 105 markers in 21 sub-populations.

**Table S2.** Pairwise *F_ST_* and *D_EST_* values between 21 breeds.
