# Supplementary material for: Conservation priorities of genetic diversity in domesticated metapopulations: a study in taurine cattle breeds
Source: Ecol Evol. 2011 Nov;1(3):408–20. doi: 10.1002/ece3.39 (PMC3287311; doi:10.1002/ece3.39)
Supplement: Supplementary file 1 [file ece30001-0408-SD1.doc]

**Supplement information:**

**A**

The sampling region of the nine Buša strains is shown in Figure S1. All strains are marked with a different colour.

SLOVENIA

CROATIA

HUNGARY

ROMANIA

SERBIA

KOSOVO-

UNMIK

MONTE-

NEGRO

BOSNIA

AND

HERZEGOVINA

ALBANIA

MACEDONIA

*Prespa*

*Lake*

GREECE

BULGARIA

ADRIATIC

SEA

AEGEAN

SEA

**RMB**

**MBU**

**ILB**

**IMB**

**GGB**

**BHB**

**HRB**

**MNB**

**PRB**

**Figure S1:** Map of the Balkan area with the origin of the Buša strains. Croatian Buša (HRB) green, Bosnian-Herzegovinian Buša (BHB) yellow, Gray Gacko Buša (GGB) blue, Montenegrin Buša (MNB) dark blue, Red Metohian Buša (RMB) red, Macedonian Buša (MBU) light blue, Illyrian Lowland Buša (ILB) light orange, Illyrian Mountain Buša (IMB) dark orange and Prespa Cattle (PRB) violet.

**Detailed description of newly analysed Buša** **strains Bosnian-Herzegovinian Buša (BHB), Montenegrin Buša (MNB) and Prespa Cattle (PRB):**

**Detailed description of BHB:**

Bosnian-Herzegovinian Buša cattle is a small native breed distributed over the large mountainous and rocky areas of Bosnia and Herzegovina (Figure S1), and are well adapted to difficult environmental conditions over centuries. There are similarities with other Buša types in the Balkan region (Medugorac *et al.* 2009). The average cow stands 104 cm and weighs 240 kg (Figure S2A), while the average bull measures 115 cm and weighs 350 kg (Figure S2B). The animals are mostly unicoloured brown with variations according to the regional strain; for example red, grey, blue-gray, black (Hrasnica *et al*. 1958). Buša heifers are late-maturing; they become sexually mature with two years. The calves are very small with a birth weight around 15 kg (Adilović & Andrijanić 2005). The breed's fertility is approximately 90 %. The life time of the cattle is about twenty years. BHB was used for milk, beef and sporadic for work. Milk production is between 800–1500 litres with 4–6% milk fat and 4.5 % protein.

**Figure S2A and B**: The BHB cow (A) and bull (B) from the Buša rezervat Buhovo (2010). Specific for the Bušas is the so-called venison snout which means a wreath of lighter hairs around a darkly pigmented snout. The head is relatively long, with fine horns curved upwards and forward with yellowish or white coloured horn basis, darker medium part and usually black top. All visible mucosa is darker colored. Udder is short, shallow and small, covered with thick white coloured hair. Lower parts of the Scrotum are darkly coloured. Hoofs are always dark-coloured, relatively broad, hard and well adapted to the rocky ground.

*Breeding area and history*:

Until the middle of the 19th century, approximately 90% of the cattle population in Bosnia and Herzegovina belonged to the indigenous short-horned Buša type. After the Bosnia and Herzegovina Annexation in 1878 from the Austria-Hungary Empire, BHB was crossed in some areas with an Austrian breed, mainly with bulls of Tyrolean Gray, Braunvieh and Fleckvieh. Since 1887 there exists a breeding centre in Gacko for Gatačko cattle (GGB). Gatačko cattle is a crossbreed between two breeds, domestic Buša cows and Alpine Gray bulls (e.g. Wipptaler and Oberinntaler ancestor strains of TGV).

Today, only low numbers of origin BHB can be found in isolated villages with poor infrastructure and without possibilities for artificial insemination.

*Current status, breeding programme and sampling*:

In 2008, the total population size was approximately 250 living animals in the area of the Federation of Bosnia and Herzegovina and 500 in the Republic of Srpska. The total population size for GGB was around 8000-12000. The animals are held locally, without selection and breeding organization.

In total, 52 blood samples were collected from BHB, which were kept on several farms in south, middle and north-western Bosnia (Figure S1). The sampling area of BHB is clearly distanced to sampling and breeding area of GGB which is systematically upgraded by TGV (see above). Written and verbal information were used to ensure the sampling of purebred Buša as much as possible. The blood samples for the analysis were sampled between February and June 2008.

**Detailed description of MNB:**

The main features of the Balkan Buša breed described in Medugorac *et al*. (2009) and Ramljak *et al.* (2011) are also resembled by the Montenegrin Buša (MNB). The body size is small, around 110 cm wither’s height, the weight is usually around 150 to 250 kg, but can reach 300 kg if the husbandry is optimized. The hair is long and usually single-coloured of black, red, dark or light brown. Multicoloured more or less striped animals are called Tiger Buša. Figures S3A to C present typical MNB animals from our sampling area. Figure S3D presents one atypical multicoloured, striped animal. The horn of the feet is strong and usually dark-pigmented (Adametz 1925, Lalatović 1957, Ljumović 1961, 1964, Marković *et al*. 2007)*.* The horns are thin and bended forward in the form of a wreath or a pitchfork. The breed matures late and shows a long production life (10 to 15 lactations and more are common). It is well adapted to the very harsh feeding and housing conditions and resistant to diseases. The latest studies showed that the body size is slightly increasing probably due to improved husbandry conditions.

*Breeding area and history*:

Until the middle of the 20th century, approximately 95% of the cattle population in Montenegro belonged to the Buša type. During the second half of the 20th century, cross-breeding was started in some areas, mainly with ancestors of Tyrolean Gray, Braunvieh and Fleckvieh .

**Figure S3A-D:** Montenegrin Buša cow (A) and bull (B) from the south-eastern coast (Ulcinj 2009). An unusual striped cow from the north-eastern region (C) (Nikšić 2010) and a Montenegrin bull from the north-eastern region (D) (Nikšić 2009).

Currently, purebred MNB is mainly reared in low numbers in isolated villages with poor infrastructure and far away from the urban centres and main roads: in the southern and northern east part of Montenegro, around Skadar Lake and the estuary of Bojana River in the Adriatic Sea, Lim River valley and Rožaje in the Ibar River valley (Figure S1). Eight of the 43 blood samples for the present study were sampled in the south-eastern region, while the remaining were sampled in the north-eastern region (Fig. S1).

*Production characteristics and traditional range of use*:

In the past, MNB was used in the extensive production system for milk and meat production and work. Since breeding association and recording of MNB animals do not exist, the exact statistically approved data on production traits are not available. Nevertheless, it is estimated that the milk production is good in relation to the body size and to the extensive husbandry conditions. During one lactation period, it can easily produce more than 2000 l of milk with a relatively high fat percentage. The meat production is modest due to the small body size.

*Current status, breeding programme and sampling*:

In the South-east of Montenegro, MNB is held as extensive grazing suckler cows except for the calving season in January/February when the cows are kept in barns. In the North-east mountainous part of the country the period in the barn is much longer with hay being the main feed during that period. The current (2010) population size of MNB in Montenegro is very small. According to the results of monitoring and additional estimates, the breeding animals comprise 100–200 individuals and the breed is endangered by extinction as the population size has a decreasing trend. An *in situ* program was established (2007) which included three herds with 45 breeding cows and three bulls. Although the government subsidies this program, it is still not enough encouragement for increased breeding/maintenance interest in Montenegro.

**Detailed description of Prespa Cattle (PRB)**

The Prespa Cattle looks similar to Buša and Brachyceros but is distinctively different. The head is long with a narrow nose, relatively large eyes, the size of the cows at wither is only 95-105 cm, the mucous membranes are gray-black, and the colour of the coat is gray – grayish, blue – brown – reddish, brown – ochre, sometimes dark; the hair coat of the original type is often coarse and sometimes shaggy (Figure S4A-C).

*Breeding area and history:*

In order to document the population and to discuss if it really represents an own breed or just a local type of another breed, two search tours have been conducted (Grunenfelder, 2006). The distribution area (Figure S1) is geographically isolated. Massive and high mountains form the border to neighbouring areas. The Prespa area is situated within the border region of three countries (Albania, Greece, FYR Macedonia) and was a military area for a long time. Until recently, there was little human influence from outside.

In FYR Macedonia the Prespa breed apparently lived until 2004 in a very remote area in two villages directly at the Albanian border with a stock of some 200 cattle.

The Albanian area at the Prespa Lakes has to be divided into two parts: the part at Lake Micro Prespa, opening towards the Devoll Plains. Here, the influence of foreign breeds has nearly completely swamped out the autochthonous cattle breeds. The second part is located at Lake Macro Prespa and separated from other Albanian regions by a 2500 m mountain massif. Modern high performance breeds have been introduced only recently. Of a total of 1500 cattle livestock at various locations some 300-400 were classified as pure bred by morphology. Sires of the old breed are still kept.

Using historical photo material, a dozen animals of the old Prespa type could be identified in the Greek village Psarades.

**Figure S4A-D:** Prespa cow at Liqenas, Albania (A) and a so-called „blue“ cow in the Greek Psarades (B). An older picture of a Prespa cow from Giorgios Catsadorakis (C) and a dwarf Prespa cow again at Liqenas, Albania (D).

*Production characteristics and traditional range of use:*

Breeding performance is not known, the local breed is optimally adapted to local conditions and performs according to expectations without much input. Although there does not exist any monitoring data it is to be expected that the breed’s input-output relation is very good and due to its adaptation it has an excellent suitability for low-input agriculture. The use in nature protection is very important as the Prespa area is a region of outstanding importance for international nature protection (amongst other Ramsar wetlands).

*Current status and breeding programme and sampling:*

In 2007, a conservation project started limited to the village of Liqenas with tagging and registering of purebred animals including a bull management. Samples were taken and 50 of them were used for the current study. Since then the breeding herd could be enlarged from 54 to 82 registered animals until 2010. The combination of agriculture and nature protection (protection/use) within a networking of agrobiodiversity, nature protection and rural development includes the protection of Prespa cattle.

**Supplement Figure S5:** Presentation of Nei`s *D_A_*-distance. **A** Consensus Neighbour-joining tree. **B** Phylogenetic network with the program *SplitsTree4* (Huson & Bryant 2006).

**Recent divergent selection but similar historical background of Holstein Frisian dairy cattle and Blanc-Bleu Belge cattle:**

The Holstein Frisian dairy cattle (HF, Figure S6A) and Blanc-Bleu Belge cattle (BBB, Figure S6B) are today understood as the most obvious phenotypic opponents. Red Holstein is understood as a red-coloured subpopulation of HF. All HF, RH and BBB have the same geographical origin (Figure S6C) and the double-muscled phenotype, now typical for BBB, segregated in both HF and BBB at the beginning of the twentieth century (Kronacher 1934). The very recent divergent selection resulted in an enormous morphological differentiation between HF and BBB, but the genetic background is highly similar as represented by measures of the neutral genetic differentiation between RH and BBB (both *G’_ST_* and *D_EST_*) based on microsatellite allele frequencies (Table S2). The average $\overline{G'}_{ST}$ and $\overline{D}_{EST}$–value between BBB and alpine and north-western breeds ($\overline{G'}_{ST}$ = 0.0905; $\overline{D}_{EST}$ = 0.123) is significantly higher than between phenotypic opponents BBB and RH (*F_ST_* = 0.0614; *D_EST_* = 0.077). The close similarity of the common background is also represented by a low genetic distance reflected by the long common evolutionary branch in both Consensus Neighbour-joining tree (Figure S5A) and Phylogenetic network (Figure S5B).


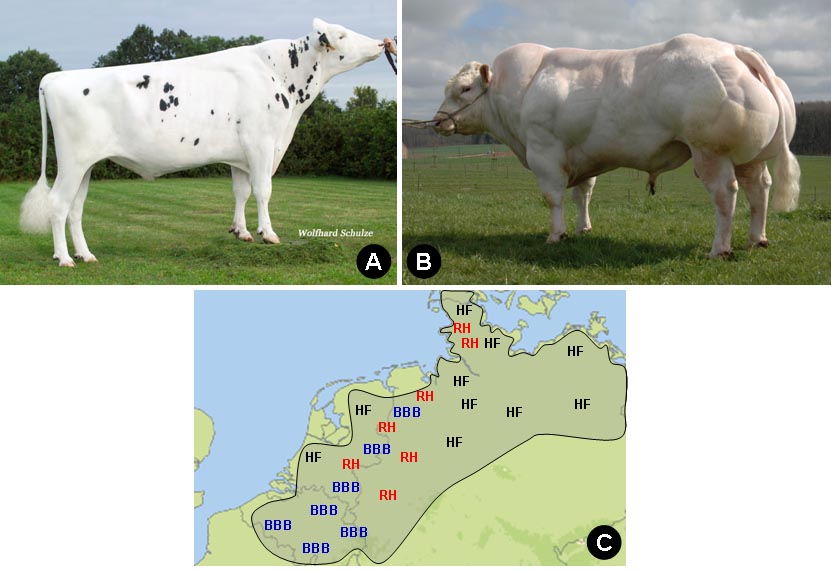


**Supplement Figure S6A-C:** The phenotypic opponents Holstein Frisian dairy cattle (A) and Blanc-Bleu Belge cattle (B, kindly provided by Alexandre Osio from the Belgian Blue Group). Despite the morphological differences they share the same geographical origin (C).

**References:**

Adametz L. (1925) Untersuchungen über den Schädelbau des Brachyceros-Rindes aus dem Polje von Podgorica (Süd-Montenegro, S.H.S). *Zeitschrift für Tierzüchtung und Züchtungsbiologie einschließlich Tierernährung*, **3**: 209-221.

Adilović S., Andrijanić M. (2005) “Bosansko-hercegovačke autohtone pasmine domaćih životinja”. Veterinarski fakultet Sarajevo.

Grunenfelder, H.-P. (2006) Prespa cattle: Identification and possible conservation measures. http://www.save-foundation.net/english/PDF/Prespa_cattle.pdf.

Hrasnica F., Ilančić D., Pavlović S., Rako A., Šmalcelj I. (1958) Specijalno stočarstvo. Poljoprivredni nakladni zavod. 612.

Huson D.H., Bryant D. 2006. Application of phylogenetic networks in evolutionary studies. *Journal of Molecular Biology and Evolution* **23**: 254-267.

Kronacher C. (1934) Genetik und Tierzüchtung. Verlag Gebrüder Borntraeger, Berlin, pp138.

Lalatović P. (1957) Tjelesna gradja i mliječnost domaće buše bivših SZR-a u Budimlju, Buču i Lušcu kod Ivangrada. *Stočarstvo*, **1-2:** 44-56.

Ljumović M. (1963) Prilog poznavanju klaničnih vrijednosti goveda sjevernog područja Crne Gore. *Naša poljoprivreda i šumarstvo* **3**: 41-50.

Ljumović M. (1964) Prilog poznavanju nasljednih i ambijentalnih varijacija – tipova goveda Polimlja. *Doctor Thesis*. Titograd, Jugoslavia.

Marković B., Marković M., Adzić N. (2007) The farm animal genetic resources of Montenegro. *Biotechnology in Animal Husbandry*, **23:** 1 - 9.

Medugorac I., Medugorac A., Russ I., Veit-Kensch C.E., Taberlet P., Luntz B., Mix H.M., Förster M. (2009) Genetic diversity of European cattle breeds highlights the conservation value of traditional unselected breeds with high effective population size. *Molecular Ecology* **18**: 3394-3410

Ramljak J., Ivanković A., Veit-Kensch C.E., Förster M., Medugorac I. (2011) Analysis of genetic and cultural conservation value of three indigenous Croatian cattle breeds in a local and global context. *Journal of Animal Breeding and Genetics* **128**:73-84.
