# Supplementary material for: Conservation priorities of genetic diversity in domesticated metapopulations: a study in taurine cattle breeds
Source: Ecol Evol. 2011 Nov;1(3):408–20. doi: 10.1002/ece3.39 (PMC3287311; doi:10.1002/ece3.39)
Supplement: Supplementary file 2 [file ece30001-0408-SD2.doc]

**Table S1:** Summary information of 105 markers in 21 subpopulations (965 individuals): Marker name (Locus), chromosome (Chr), relative position (cM), number of alleles (*nA*), expected and observed heterozygosity (*H_E_* and *H_o_*) and possible reason for marker exclusion (Commentary). The 30 microsatellite markers recommended by the ISAG/FAO working group (http://dad.fao.org/) are marked grey.

| **Locus:** | **Chr** | **cM** | **nA** | ***H_E_*** | ***Ho*** | **Commentary** |
| --- | --- | --- | --- | --- | --- | --- |
| BM6438 | 01 | 1.781 | 11 | 0.426 | 0.374 | Neutral |
| ILSTS004 | 01 | 34.340 | 12 | 0.512 | 0.407 | Decisive evidence for directional selection |
| BMS4024 | 01 | 45.252 | 19 | 0.876 | 0.784 | Neutral |
| BM1312 | 01 | 61.852 | 10 | 0.649 | 0.590 | Neutral |
| DVEPC56 | 01 | 70.988 | 11 | 0.760 | 0.724 | Neutral |
| BM6506 | 01 | 77.686 | 11 | 0.573 | 0.497 | Decisive evidence for directional selection |
| DIK4781 | 01 | 80.044 | 15 | 0.814 | 0.781 | Neutral |
| DIK5103 | 01 | 85.428 | 17 | 0.682 | 0.630 | Neutral |
| DIK4151 | 01 | 89.989 | 11 | 0.752 | 0.679 | Neutral |
| BMS4010 | 01 | 98.053 | 7 | 0.720 | 0.686 | Neutral |
| BM864 | 01 | 99.714 | 23 | 0.762 | 0.725 | Neutral |
| DIK4491 | 01 | 106.641 | 8 | 0.515 | 0.497 | Decisive evidence for directional selection |
| BMS1789 | 01 | 113.501 | 13 | 0.828 | 0.772 | Neutral |
| BMS1939 | 01 | 117.796 | 13 | 0.625 | 0.550 | Neutral |
| BMS4041 | 01 | 119.817 | 7 | 0.567 | 0.511 | Neutral |
| BM1824 | 01 | 122.391 | 9 | 0.757 | 0.710 | Neutral |
| CSSM19 | 01 | 122.094 | 11 | 0.761 | 0.679 | Neutral |
| UWCA46 | 01 | 127.441 | 15 | 0.837 | 0.781 | Neutral |
| DIK126 | 01 | 130.041 | 15 | 0.653 | 0.564 | Decisive evidence for directional selection |
| DIK125 | 01 | 130.325 | 8 | 0.715 | 0.641 | Neutral |
| MAF46 | 01 | 132.471 | 5 | 0.409 | 0.367 | Neutral |
| BMS918 | 01 | 132.471 | 10 | 0.807 | 0.761 | Neutral |
| BMS4044 | 01 | 142.244 | 15 | 0.877 | 0.797 | Neutral |
| DIK2050 | 01 | 144.391 | 9 | 0.665 | 0.451 | Decisive evidence for directional selection |
| BMS4014 | 01 | 148.212 | 12 | 0.794 | 0.707 | Neutral |
| DIK2040 | 01 | 152.189 | 9 | 0.742 | 0.704 | Neutral |
| ILSTS026 | 02 | 10.772 | 9 | 0.613 | 0.569 | Neutral |
| TGLA377 | 02 | 30.736 | 15 | 0.676 | 0.603 | Neutral |
| ILSTS030 | 02 | 38.901 | 7 | 0.645 | 0.610 | Neutral |
| BMS803 | 02 | 44.514 | 14 | 0.750 | 0.681 | Neutral |
| ILSTS098 | 02 | 60.259 | 18 | **0.615** | **0.474** | More than half of subpopulations not in HWE |
| TEXAN01 | 02 | 74.547 | 12 | 0.694 | 0.621 | Neutral |
| BMS1866 | 02 | 88.761 | 13 | 0.833 | 0.786 | Neutral |
| BM2113 | 02 | 115.437 | 11 | 0.853 | 0.776 | Neutral |
| BMS871 | 03 | 0.000 | 8 | 0.392 | 0.340 | Neutral |
| INRA006 | 03 | 17.088 | 12 | 0.537 | 0.489 | Neutral |
| BMS482 | 03 | 34.038 | 13 | 0.755 | 0.686 | Neutral |
| INRA023 | 03 | 65.000 | 13 | 0.819 | 0.742 | Neutral |
| INRA003 | 03 | 59.360 | 14 | 0.682 | 0.630 | Neutral |
| HUJ246 | 03 | 67.982 | 10 | 0.803 | 0.730 | Neutral |
| HUJII77 | 03 | 87.331 | 12 | 0.804 | 0.757 | Neutral |
| IDVGA35 | 03 | 102.539 | 16 | 0.865 | 0.828 | Neutral |
| BMC4214 | 03 | 125.802 | 14 | 0.794 | 0.742 | Neutral |
| BMS1788 | 04 | 12.544 | 12 | 0.857 | 0.783 | Neutral |
| RM067 | 04 | 51.928 | 11 | 0.790 | 0.710 | Neutral |
| BM1224 | 04 | 54.835 | 12 | 0.854 | 0.749 | Neutral |
| BR6303 | 05 | 104.912 | 7 | 0.736 | 0.669 | Neutral |
| ETH10 | 05 | 71.764 | 9 | 0.715 | 0.654 | Neutral |
| ETH152 | 05 | 121.749 | 11 | 0.755 | 0.724 | Neutral |
| DIK4224 | 06 | 0.835 | 13 | 0.742 | 0.683 | Neutral |
| **Locus:** | **Chr** | **Position** | **nA** | ***H_E_*** | ***Ho*** | **Commentary** |
| MNB207 | 06 | 3.488 | 17 | 0.649 | 0.616 | Neutral |
| INRA133 | 06 | 8.053 | 11 | 0.591 | 0.477 | Neutral |
| DIK4408 | 06 | 9.028 | 12 | **0.847** | **0.653** | More than half of subpopulations not in HWE |
| DIK5285 | 06 | 15.362 | 10 | 0.716 | 0.671 | Neutral |
| DIK2029 | 06 | 16.010 | 13 | 0.538 | 0.500 | Neutral |
| BM1329 | 06 | 35.398 | 11 | 0.752 | 0.726 | Neutral |
| BMS1242 | 06 | 52.842 | 13 | 0.771 | 0.718 | Neutral |
| BM143 | 06 | 53.724 | 14 | 0.798 | 0.731 | Neutral |
| DIK082 | 06 | 57.566 | 11 | **0.727** | **0.593** | More than half of subpopulations not in HWE |
| BM4322 | 06 | 63.865 | 22 | 0.593 | 0.320 | Decisive evidence for directional selection |
| BMS360 | 06 | 72.882 | 18 | 0.793 | 0.721 | Neutral |
| OAREL03 | 06 | 88.178 | 10 | 0.776 | 0.712 | Neutral |
| CSN3 | 06 | 89.354 | 8 | 0.543 | 0.476 | Neutral |
| BP7 | 06 | 98.496 | 9 | 0.648 | 0.568 | Neutral |
| BM8124 | 06 | 101.408 | 14 | 0.518 | 0.464 | Neutral |
| BMC4203 | 06 | 119.048 | 12 | 0.687 | 0.654 | Neutral |
| BM2320 | 06 | 127.264 | 15 | 0.747 | 0.694 | Neutral |
| BL1038 | 06 | 129.985 | 11 | 0.543 | 0.496 | Neutral |
| BMS5002 | 06 | 130.781 | 14 | 0.659 | 0.614 | Neutral |
| ILSTS006 | 07 | 116.629 | 14 | 0.785 | 0.704 | Neutral |
| HEL9 | 08 | 84.844 | 14 | 0.755 | 0.704 | Neutral |
| ETH225 | 09 | 12.754 | 11 | 0.778 | 0.699 | Neutral |
| MM12 | 09 | 84.258 | 14 | 0.816 | 0.761 | Neutral |
| CSRM60 | 10 | 77.816 | 14 | 0.742 | 0.683 | Neutral |
| INRA037 | 10 | 79.007 | 20 | **0.670** | **0.471** | More than half of subpopulations not in HWE |
| ILSTS005 | 10 | 107.964 | 7 | 0.430 | 0.392 | Decisive evidence for directional selection |
| INRA032 | 11 | 68.679 | 14 | 0.677 | 0.640 | Neutral |
| HEL13 | 11 | 122.370 | 9 | 0.674 | 0.593 | Neutral |
| INRA005 | 12 | 86.853 | 5 | 0.647 | 0.567 | Neutral |
| RM327 | 13 | 73.638 | 15 | 0.810 | 0.756 | Neutral |
| AGLA232 | 13 | 91.379 | 18 | 0.874 | 0.826 | Neutral |
| CSSM66 | 14 | 5.125 | 12 | 0.823 | 0.713 | Neutral |
| ILSTS011 | 14 | 25.708 | 8 | 0.632 | 0.607 | Neutral |
| NCAM | 15 | 22.088 | 10 | 0.650 | 0.579 | Neutral |
| HEL1 | 15 | 37.962 | 10 | 0.730 | 0.657 | Neutral |
| TGLA53 | 16 | 38.547 | 19 | 0.885 | 0.819 | Neutral |
| INRA035 | 16 | 89.000 | 10 | 0.374 | 0.341 | Neutral |
| ETH185 | 17 | 54.709 | 17 | 0.772 | 0.715 | Neutral |
| INRA063 | 18 | 47.953 | 10 | 0.633 | 0.607 | Neutral |
| TGLA227 | 18 | 84.087 | 15 | 0.872 | 0.841 | Neutral |
| ETH3 | 19 | 90.043 | 11 | 0.710 | 0.671 | Neutral |
| TGLA126 | 20 | 31.866 | 9 | 0.723 | 0.668 | Neutral |
| HEL5 | 21 | 13.520 | 12 | 0.806 | 0.722 | Neutral |
| TGLA122 | 21 | 62.685 | 25 | 0.807 | 0.744 | Neutral |
| ILSTS054 | 21 | 65.845 | 10 | 0.787 | 0.746 | Neutral |
| HAUT24 | 22 | 66.102 | 15 | 0.800 | 0.730 | Neutral |
| BM1818 | 23 | 58.195 | 12 | 0.709 | 0.655 | Neutral |
| BM7151 | 24 | 8.152 | 12 | 0.834 | 0.769 | Neutral |
| DIK2706 | 24 | 68.466 | 15 | 0.842 | 0.799 | Neutral |
| ILSTS102 | 25 | 7.199 | 13 | 0.800 | 0.738 | Neutral |
| AF5 | 25 | 61.669 | 14 | 0.816 | 0.746 | Neutral |
| HAUT27 | 26 | 35.189 | 11 | 0.790 | 0.683 | Neutral |
| BM3507 | 27 | 0.000 | 18 | 0.801 | 0.671 | Neutral |
| CSSM36 | 27 | 43.002 | 13 | 0.784 | 0.749 | Neutral |
| IDVGA43 | 28 | 34.999 | 4 | **0.545** | **0.305** | More than half of subpopulations not in HWE |

**Supplement Table S2:** Pair-wise *F_ST_* (represented by nearly unbiased estimator *G_ST_’*, Nei & Chesser (1983), below diagonal) and *D_EST_* (harmonic mean of Jost (2007) estimator of true differentiation (Crawford 2010), above diagonal) between 21 breeds. Two breeds with known population bottlenecks HRP and GLW show high *F_ST_* and *D_EST_* values in comparison with all breeds (*F_ST_* > 0.071, $\overline{F_{ST}}$ = 0.119; *D_EST_* > 0.108, $\overline{D_{EST}}$ = 0.174). Both *F_ST_* and *D_EST_* estimates are high within the north-western European breeds ($\overline{F_{ST}}$ = 0.085; $\overline{D_{EST}}$ = 0.124), alpine breeds are within a medium level ($\overline{F_{ST}}$ = 0.069; $\overline{D_{EST}}$ = 0.083), while nine Buša breeds displayed very low *F_ST_* and $\overline{D_{EST}}$ values to each other ($\overline{F_{ST}}$ = 0.016; $\overline{D_{EST}}$ = 0.021) and relatively high *F_ST_* and *D_EST_* values to non-Buša breeds ($\overline{F_{ST}}$ = 0.060; $\overline{D_{EST}}$ = 0.092).

| **Pop** | **MBU** | **PRB** | **IMB** | **ILB** | **RMB** | **MNB** | **BHB** | **GGB** | **HRB** | **HRP** | **HRI** | **TGV** | **OBV** | **MWF** | **AMB** | **FGV** | **FV** | **TAR** | **RH** | **BBB** | **GLW** | $\overline{D_{EST}}$ |
| --- | --- | --- | --- | --- | --- | --- | --- | --- | --- | --- | --- | --- | --- | --- | --- | --- | --- | --- | --- | --- | --- | --- |
| **MBU** | 0 | 0.032 | 0.029 | 0.010 | 0.010 | 0.006 | 0.025 | 0.017 | 0.012 | 0.172 | 0.091 | 0.087 | 0.052 | 0.084 | 0.075 | 0.086 | 0.067 | 0.082 | 0.089 | 0.088 | 0.138 | 0.063 |
| **PRB** | 0.023 | 0 | 0.032 | 0.017 | 0.022 | 0.018 | 0.034 | 0.037 | 0.019 | 0.196 | 0.103 | 0.099 | 0.069 | 0.103 | 0.091 | 0.097 | 0.088 | 0.108 | 0.087 | 0.104 | 0.118 | 0.074 |
| **IMB** | 0.021 | 0.024 | 0 | 0.012 | 0.022 | 0.029 | 0.032 | 0.042 | 0.025 | 0.198 | 0.099 | 0.097 | 0.077 | 0.104 | 0.086 | 0.105 | 0.092 | 0.100 | 0.100 | 0.096 | 0.126 | 0.075 |
| **ILB** | 0.011 | 0.020 | 0.013 | 0 | 0.007 | 0.011 | 0.029 | 0.015 | 0.011 | 0.175 | 0.097 | 0.080 | 0.049 | 0.080 | 0.051 | 0.080 | 0.059 | 0.100 | 0.084 | 0.089 | 0.109 | 0.058 |
| **RMB** | 0.008 | 0.020 | 0.016 | 0.010 | 0 | 0.012 | 0.026 | 0.027 | 0.012 | 0.184 | 0.076 | 0.094 | 0.062 | 0.086 | 0.078 | 0.091 | 0.072 | 0.089 | 0.069 | 0.083 | 0.123 | 0.062 |
| **MNB** | 0.009 | 0.017 | 0.019 | 0.011 | 0.010 | 0 | 0.022 | 0.018 | 0.004 | 0.174 | 0.083 | 0.063 | 0.031 | 0.074 | 0.064 | 0.063 | 0.047 | 0.074 | 0.062 | 0.067 | 0.112 | 0.052 |
| **BHB** | 0.019 | 0.028 | 0.023 | 0.020 | 0.017 | 0.016 | 0 | 0.029 | 0.022 | 0.178 | 0.095 | 0.082 | 0.068 | 0.098 | 0.080 | 0.080 | 0.082 | 0.100 | 0.093 | 0.105 | 0.124 | 0.070 |
| **GGB** | 0.015 | 0.028 | 0.028 | 0.016 | 0.020 | 0.011 | 0.021 | 0 | 0.013 | 0.197 | 0.104 | 0.049 | 0.036 | 0.071 | 0.070 | 0.077 | 0.050 | 0.093 | 0.075 | 0.086 | 0.117 | 0.061 |
| **HRB** | 0.011 | 0.019 | 0.018 | 0.010 | 0.010 | 0.006 | 0.018 | 0.010 | 0 | 0.176 | 0.077 | 0.068 | 0.039 | 0.072 | 0.062 | 0.076 | 0.052 | 0.078 | 0.074 | 0.078 | 0.114 | 0.054 |
| **HRP** | 0.109 | 0.119 | 0.120 | 0.112 | 0.102 | 0.108 | 0.112 | 0.126 | 0.109 | 0 | 0.204 | 0.225 | 0.232 | 0.246 | 0.217 | 0.239 | 0.220 | 0.221 | 0.224 | 0.230 | 0.213 | **0.206** |
| **HRI** | 0.052 | 0.060 | 0.058 | 0.055 | 0.043 | 0.049 | 0.057 | 0.060 | 0.048 | 0.132 | 0 | 0.135 | 0.136 | 0.148 | 0.131 | 0.141 | 0.145 | 0.128 | 0.125 | 0.157 | 0.159 | 0.122 |
| **TGV** | 0.054 | 0.066 | 0.066 | 0.054 | 0.058 | 0.046 | 0.057 | 0.036 | 0.045 | 0.162 | 0.094 | 0 | 0.069 | 0.107 | 0.098 | 0.099 | 0.095 | 0.118 | 0.139 | 0.142 | 0.145 | 0.104 |
| **OBV** | 0.038 | 0.045 | 0.047 | 0.038 | 0.040 | 0.025 | 0.044 | 0.028 | 0.029 | 0.156 | 0.085 | 0.054 | 0 | 0.073 | 0.072 | 0.074 | 0.057 | 0.075 | 0.105 | 0.106 | 0.146 | 0.082 |
| **MWF** | 0.053 | 0.064 | 0.064 | 0.052 | 0.054 | 0.045 | 0.058 | 0.046 | 0.044 | 0.163 | 0.094 | 0.077 | 0.051 | 0 | 0.103 | 0.104 | 0.091 | 0.098 | 0.125 | 0.136 | 0.205 | 0.110 |
| **AMB** | 0.049 | 0.060 | 0.056 | 0.040 | 0.052 | 0.042 | 0.051 | 0.048 | 0.041 | 0.152 | 0.089 | 0.070 | 0.055 | 0.073 | 0 | 0.034 | 0.059 | 0.086 | 0.126 | 0.124 | 0.139 | 0.092 |
| **FGV** | 0.059 | 0.067 | 0.069 | 0.055 | 0.061 | 0.048 | 0.055 | 0.054 | 0.049 | 0.167 | 0.099 | 0.073 | 0.061 | 0.075 | 0.034 | 0 | 0.055 | 0.100 | 0.119 | 0.125 | 0.148 | 0.100 |
| **FV** | 0.051 | 0.058 | 0.060 | 0.050 | 0.052 | 0.040 | 0.055 | 0.044 | 0.038 | 0.156 | 0.093 | 0.069 | 0.049 | 0.070 | 0.049 | 0.051 | 0 | 0.072 | 0.119 | 0.100 | 0.138 | 0.088 |
| **TAR** | 0.058 | 0.065 | 0.068 | 0.062 | 0.060 | 0.051 | 0.066 | 0.057 | 0.052 | 0.160 | 0.095 | 0.087 | 0.063 | 0.078 | 0.070 | 0.081 | 0.059 | 0 | 0.157 | 0.127 | 0.177 | 0.109 |
| **RH** | 0.062 | 0.059 | 0.066 | 0.060 | 0.052 | 0.047 | 0.064 | 0.057 | 0.052 | 0.153 | 0.086 | 0.097 | 0.073 | 0.082 | 0.088 | 0.093 | 0.087 | 0.111 | 0 | 0.077 | 0.139 | 0.109 |
| **BBB** | 0.057 | 0.062 | 0.061 | 0.057 | 0.054 | 0.048 | 0.064 | 0.058 | 0.050 | 0.156 | 0.098 | 0.096 | 0.074 | 0.086 | 0.086 | 0.093 | 0.083 | 0.095 | 0.061 | 0 | 0.157 | 0.114 |
| **GLW** | 0.083 | 0.081 | 0.084 | 0.077 | 0.077 | 0.071 | 0.085 | 0.078 | 0.075 | 0.168 | 0.111 | 0.112 | 0.099 | 0.127 | 0.110 | 0.115 | 0.109 | 0.126 | 0.109 | 0.112 | 0 | **0.142** |
| $\overline{F_{ST}}$ | 0.042 | 0.049 | 0.049 | 0.041 | 0.041 | 0.036 | 0.046 | 0.042 | 0.037 | **0.137** | 0.078 | 0.074 | 0.058 | 0.073 | 0.066 | 0.073 | 0.066 | 0.078 | 0.078 | 0.078 | **0.100** |  |

Crawford N.G. (2010) *Molecular Ecology Resources* **10**, 556–557.

Jost L (2008) *Molecular Ecology* **17**, 4015–4026.

Nei M, Chesser RK (1983) *Annals of Human Genetics* **47**, 253–259.
